# Development and Preliminary Validation of College Students’ Perceived Public Health Crisis Scale

**DOI:** 10.3390/bs16060927

**Published:** 2026-06-05

**Authors:** Cheng Cheng, Qingling Wang, Xiao Chen

**Affiliations:** 1 School of Nursing, Fudan University, Shanghai 200032, China; corollachen_cx@fudan.edu.cn; 2 School of Nursing and Health Management, Shanghai University of Medicine & Health Sciences, Shanghai 201318, China; wangql@sumhs.edu.cn

**Keywords:** crisis perception, health concerns, college students, factor analysis, scale validation

## Abstract

Public health crises may profoundly affect college students, but there is a lack of instruments specifically designed to assess their perceptions of such events. This study aimed to develop and preliminarily validate the College Students’ Perceived Public Health Crisis Scale (CSPHCS), a measure designed to assess college students’ perceptions of public health crises. The scale development followed a multistage process including item generation through literature review and student interviews, expert review, cognitive interviewing, pilot testing, and psychometric evaluation using exploratory and confirmatory factor analyses. Data were collected using an electronic questionnaire administered to college students. Exploratory factor analysis supported a three-factor, 16-item structure comprising: (1) perceived impact and personal anxiety, (2) perceived likelihood of crisis occurrence and infection, and (3) confidence in preparedness and information trust. Confirmatory factor analysis provided partial support for the three-factor model, with acceptable RMSEA and SRMR but CFI and TLI slightly below conventional thresholds. Internal consistency was acceptable for the overall scale and its subscales (Cronbach’s α = 0.758–0.804). The CSPHCS provides preliminary evidence of content validity, internal consistency, and construct validity as a measure of college students’ perceptions of public health crises. Further studies are needed to refine the scale and examine its stability, predictive utility, and applicability across diverse contexts.

## 1. Introduction

A “crisis” in healthcare refers to a critical point in the progression of a disease where a pivotal change occurs, leading either to recovery or death (Runciman & Merry, 2005). A public health crisis (e.g., COVID-19) is a challenging or complex situation that impacts healthcare systems across one or more regions, or that begins in a single location and expands worldwide (Zhu, 2023). In recent decades, public health crises, ranging from emerging infectious diseases to environmental disasters, have become more frequent and complex, presenting significant challenges to societal well-being (Hartley & Perencevich, 2020). These crises disrupt daily life, strain healthcare systems, and cause widespread psychological distress. For example, a study using longitudinal national data found that psychological distress in the US sharply increased during the early COVID-19 crisis but returned to baseline levels by mid-2020 across all sociodemographic groups, including those with pre-existing mental health conditions (Daly & Robinson, 2021). Among the affected populations, college students are a particularly vulnerable group because of their developmental stage, academic pressures, transitional life circumstances, and often limited access to comprehensive support systems (Chen et al., 2024; Wang et al., 2021). The convergence of these factors not only increases their exposure to stressors during crises but also amplifies the potential negative impacts on their educational and psychosocial development.

Understanding how college students perceive and cope with public health crises is therefore of paramount importance. Perception is a fundamental element in various theoretical frameworks used in mental health research (Qicheng et al., 2024). The Health Belief Model, a widely accepted approach to understanding health-related decision-making, suggests that individuals’ actions regarding their health are shaped by their perceptions of susceptibility, severity, benefits, and barriers (Abraham & Sheeran, 2007). More generally, theoretical models, such as Lazarus and Folkman’s (1984) transactional model of stress and coping, emphasize the role of cognitive appraisal in stress perception, suggesting that an individual’s evaluation of a stressful event significantly informs their coping strategies. Perceptions comprising both cognitive and emotional aspects play a significant role in shaping coping strategies, which in turn affect mental health outcomes and academic performance (Lazarus & Folkman, 1984). However, despite the critical need to elucidate these perceptions, current research has been hampered by the absence of a dedicated, empirically validated instrument tailored to this population. Existing studies have often relied on generalized measures or instruments designed for broader demographics, thereby failing to capture the nuanced experiences of college students under crisis conditions.

A substantial body of literature underscores the multifaceted impacts of public health crises on individuals and communities. Numerous studies have employed various risk perception assessment tools; however, systematic reviews have identified several limitations within these instruments. For example, Dai et al. (2020) developed a public risk perception scale for health crises based on expert consultations and an extensive literature review. They validated this scale through an online survey of 1082 participants during an influenza outbreak in China, resulting in a 10-item measure encompassing dimensions of severity, controllability, health impact, and epidemic likelihood, which demonstrated strong reliability and validity. Similarly, the COVID-19 Risk Perception Scale (CoRP) was designed to evaluate individuals’ perceived seriousness of COVID-19, the likelihood of infection for themselves or close contacts within six months, and their current level of worry (Capone et al., 2021). Shen et al. (2021) developed a risk perception scale for public health emergencies and evaluated its validity and reliability during the COVID-19 pandemic. Through a questionnaire survey of 504 Chinese adults, they identified a nine-item scale that demonstrated satisfactory psychometric properties. Motta Zanin et al. (2020) used the Public Risk Perception questionnaire to examine Italians’ perceptions of health risks during the pandemic. In related research, Zhang et al. (2022) developed the Haze Risk Perception Scale (HRPS), which comprises four dimensions ordered hierarchically according to the degree of perceived risk: direct consequences perception, indirect consequences perception, risk responsibility perception, and risk source perception. Additionally, the Haze Risk Perception Influencing Factor Scale (HRPIFS) consists of three distinct dimensions. Moreover, Graffigna et al. (2021) developed and validated the Public Health Engagement Scale for Emergency Settings (PHEs-E), a psychometric instrument designed to assess individuals’ readiness to adopt behavioral changes necessary for mitigating public health crises.

Despite these valuable insights, limitations remain in the current measurement tools. Most existing scales are not specifically designed for college students and often lack the precision needed to capture the unique cognitive responses of this subgroup during a public health crisis. The development of the College Students’ Perceived Public Health Crisis Scale (CSPHCS) is informed by this gap and aims to address it. This instrument not only enriches the existing literature but also serves as a vital resource for designing targeted interventions and policies to promote resilience and mitigate the adverse effects of public health crises on this vulnerable population.

## 2. Aims

The present study aimed to develop the College Students’ Perceived Public Health Crisis Scale and to examine its preliminary psychometric properties in a sample of college students.

## 3. Methods

**Figure 1:**
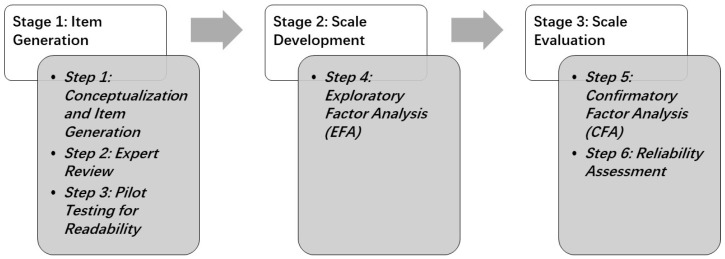
Flowchart of the development and validation process of the CSPHCS.

This study utilized a multistage instrument development design (Hunter & Brewer, 2015), integrating qualitative and quantitative approaches to facilitate a comprehensive scale development process. The development and validation of the CSPHCS followed a systematic, multi-stage procedure, including item generation, refinement, pilot testing, and rigorous validation (Boateng et al., 2018). Figure 1 illustrates the entire process of developing and validating the CSPHCS.

The development of the CSPHCS was guided by both theoretical and empirical considerations. Conceptually, the initial domains were informed by the Health Belief Model (Abraham & Sheeran, 2007) and by Lazarus and Folkman’s transactional model of stress and coping (Lazarus & Folkman, 1984), which highlights the role of cognitive appraisal and emotional response in stressful situations. In addition, previous public health risk-perception instruments were reviewed to identify commonly assessed dimensions, including perceived likelihood, perceived severity, controllability, health impact, affective risk perception, trust in information, and confidence in protective measures.

### 3.1. Stage 1: Item Generation

#### 3.1.1. Step 1: Conceptualization and Item Generation

A PRISMA-guided systematic review was conducted to identify existing instruments assessing college students’ risk perceptions during public health crises. Searches of PubMed, CINAHL Complete, EMBASE, Web of Science, and PsycArticles identified 18 scales covering dimensions such as perceived susceptibility, severity, controllability, affective and cognitive risk perception, individual and societal risk, and constructs derived from the Health Belief Model. Most studies reported acceptable reliability, typically using Cronbach’s alpha. College students showed moderate to high levels of risk perception throughout the pandemic. Their risk perceptions were associated with demographic characteristics, information-seeking behaviors, and social environmental factors. The review provided an evidence base for the preliminary development of scale items.

Subsequently, the primary author conducted a descriptive qualitative study (Doyle et al., 2020) involving 14 college students selected through convenience sampling. Semi-structured interviews, designed by the research team and verified by an experienced qualitative research expert, were employed. Inclusion criteria were: (1) full-time enrollment in an undergraduate program; (2) current sophomore, junior, or senior status; and (3) willingness and ability to articulate personal experiences and perceptions related to public health crises. Exclusion criteria included: (1) first-year students, due to limited university experience; and (2) students on academic leave or not residing on campus during the crisis period.

The mean age of participants was 19.9 years (SD = 1.1), with an equal gender distribution (50% Female). Interviews lasted approximately 30 min, were audio-recorded, and transcribed verbatim. Interview transcripts were analyzed using a descriptive qualitative content analysis approach. First, two researchers independently read the transcripts several times to become familiar with the data. Meaning units related to students’ perceptions of public health crises were identified and coded. Codes were then compared, discussed, and grouped into broader conceptual categories, such as perceived likelihood of crisis occurrence, perceived infection risk, perceived academic and social impact, emotional response, confidence in protective measures, preparedness, and trust in crisis-related information. Disagreements in coding were resolved through discussion, and unresolved discrepancies were reviewed with a qualitative research expert until consensus was reached. The categories derived from the interviews were then compared with the domains identified from the literature review. Items were generated or revised when they reflected both theoretical relevance and student-reported experiences. This process yielded a preliminary pool of 22 items reflecting a broad spectrum of risk perceptions, each phrased as a question rated on a 5-point Likert scale from 1 (very low) to 5 (very high).

The preliminary domains were therefore generated deductively from the literature and theoretical models, and then refined inductively through qualitative interviews with college students. Candidate domains were retained when they met four criteria: repeated appearance in previous instruments or theoretical frameworks; relevance to public health crisis perception; applicability to college students’ campus, academic, and social contexts; and conceptual distinctiveness from other domains. This combined deductive–inductive approach was used to ensure that the scale was both theoretically grounded and contextually meaningful for college students.

#### 3.1.2. Step 2: Expert Review

A Delphi method (Williamson, 2002) was employed to refine the initial 22-item pool. The primary author purposively contacted 15 experts via email, with 14 consenting to participate. Experts were selected based on: (1) possession of at least a research degree, such as a PhD in relevant disciplines (e.g., public health, psychology, nursing); (2) authorship of peer-reviewed publications related to scale development in psychology or mental health; and (3) a minimum of five years of academic or clinical experience. Exclusion criteria included a lack of instrument development experience.

The final panel comprised 14 experts from applied psychology and health psychology (*n* = 4), public health (*n* = 9), and school nursing (*n* = 1), all with prior scale development experience. Each expert independently evaluated items for relevance and clarity using a 4-point Likert scale and provided qualitative feedback. Experts also assessed item phrasing and suggested additional dimensions or items. An online consensus meeting was held to review expert ratings and proposed revisions, culminating in approval of the final item set. Quantitative content validity was assessed via the Content Validity Ratio (CVR), Item-level Content Validity Index (I-CVI), and Scale-level Content Validity Index (S-CVI). Items with CVR below the acceptable threshold (Lawshe, 1975) were removed. I-CVI values ranged from 0.71 to 0.89, and the S-CVI was 0.80, indicating acceptable content validity. Ten items were revised for clarity and theoretical alignment, four items were removed, and item descriptions were refined to enhance contextual relevance and conceptual precision.

To evaluate cognitive validity, the preliminary scale was administered to nine college students using cognitive interviewing techniques (Castillo-Díaz & Padilla, 2013). Inclusion criteria mirrored those of the qualitative phase, excluding first-year students to ensure adequate exposure to college-related stressors. Participants, with a mean age of 20.5 years (SD = 1.1) and predominantly female (*n* = 6), were all juniors. During interviews, participants verbalized their thought processes while responding to each item, enabling identification of ambiguous wording, interpretive inconsistencies, and misalignment with intended constructs. Items with confusing or divergent interpretations were revised or combined, resulting in a final set of 16 items demonstrating theoretical coherence and contextual appropriateness.

#### 3.1.3. Step 3: Pilot Testing for Readability

A preliminary pilot study was conducted with 25 college students meeting the same inclusion and exclusion criteria as prior stages. The objective was to assess item readability, clarity, and comprehensibility. Participants reviewed each item and provided feedback on relevance and interpretability. The average completion time and feasibility of future interview implementation were also evaluated. Participants had a mean age of 21.3 years (SD = 0.9), with 18 females; 59% were juniors or seniors. Qualitative feedback indicated general comprehension and suitability of items for the public health crisis context, though some items required rereading for clarity. Minor wording adjustments were made accordingly. The scale was completed in approximately 10 min on average. The revised version was adopted for the subsequent main survey.

### 3.2. Stage 2: Scale Development

#### Step 4: Exploratory Factor Analysis (EFA)

A survey announcement was disseminated via social networking applications, and data were collected using a structured questionnaire administered via WJX.com. A larger sample of college students, selected according to predefined inclusion and exclusion criteria, participated in the initial field test. The dataset was split into two subsets: one for Exploratory Factor Analysis (EFA) and the other for Confirmatory Factor Analysis (CFA). Data analysis was performed using SPSS for Windows 23 (SPSS Inc., Chicago, IL, USA) and R (Version 4.5.1).

The suitability of the data for factor analysis was confirmed by the Kaiser–Meyer–Olkin (KMO) measure and Bartlett’s test of sphericity (Fabrigar & Wegener, 2011). Parallel analysis (Iacobucci et al., 2022), based on common Factor Analysis (FA), was conducted to determine the optimal number of factors to retain, in conjunction with examination of the scree plot. Subsequently, EFA was performed using Principal Axis Factoring (PAF) with oblique rotation on the 16 items to extract the underlying latent factors. Factors with eigenvalues exceeding those obtained from parallel analysis were retained, and factor loadings above 0.40 were considered significant. Items were systematically removed based on predefined criteria, with the EFA model recalibrated if item deletion was needed.

### 3.3. Stage 3: Scale Evaluation

#### 3.3.1. Step 5: Confirmatory Factor Analysis (CFA)

The refined scale was administered to a separate subsample of college students to evaluate the factor structure identified through EFA. Because the CSPHCS items were measured on a 5-point Likert response format, CFA was conducted using the robust weighted least squares mean and variance adjusted (WLSMV) estimator, which is appropriate for ordinal indicators (Li, 2016). CFA assessed model adequacy using multiple goodness-of-fit indices (Goretzko et al., 2024), including the chi-square to degrees of freedom ratio (χ^2^/df), Comparative Fit Index (CFI), Tucker–Lewis Index (TLI), Root Mean Square Error of Approximation (RMSEA), and Standardized Root Mean Square Residual (SRMR). Based on commonly used recommendations, χ^2^/df values below 3 were considered acceptable. CFI and TLI values of 0.90 or above were interpreted as acceptable, and values of 0.95 or above as good. RMSEA values of 0.08 or below were considered acceptable, and values of 0.06 or below were considered good. SRMR values of 0.08 or below were considered acceptable. Given the preliminary nature of this scale development study, model fit was interpreted based on the overall pattern of fit indices rather than a single cutoff value.

#### 3.3.2. Step 6: Reliability Assessment

The reliability of the CSPHCS was evaluated through internal consistency estimates (Cronbach’s α), calculated for each subscale and the overall scale.

## 4. Results

### 4.1. Descriptive Statistics and Demographics of the Sample

Of the 599 distributed questionnaires, 564 were retained after assessing completeness and validity. The distribution of responses is shown in Table 1.

### 4.2. Factor Structure and Loadings of the CSPHCS

#### 4.2.1. EFA Results

The sample was then divided into two groups: 282 cases were randomly selected for EFA, and the remaining 282 cases were set aside for subsequent CFA. In the EFA sample, the mean age was 21.04 years, with an age range of 16 to 25 years. The sample included 111 males, 169 females, and 2 participants who preferred not to disclose their sex. In the CFA sample, the mean age was 21.36 years, with an age range of 16 to 33 years; one participant had missing age information. The CFA sample included 119 males, 159 females, and 4 participants who preferred not to disclose their sex.

The suitability of the dataset for factor analysis was confirmed by the KMO measure of sampling adequacy, which was 0.821, indicating a meritorious level of sampling adequacy. Bartlett’s test of sphericity was significant (χ^2^ = 1584.023, df = 120, *p* < 0.001), confirming that the data were appropriate for factor extraction.

**Figure 2:**
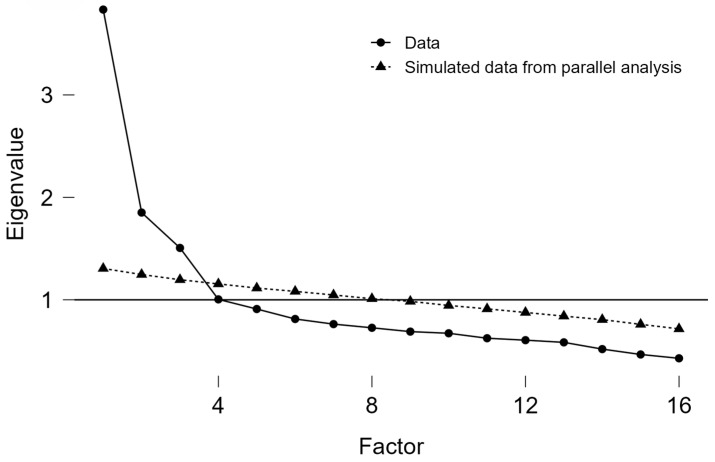
Scree plot.

Parallel analysis based on common FA suggested retaining three factors (Figure 2). Subsequently, EFA was conducted using PAF with oblique rotation on the initial 16 items. All items met the predefined loading criterion, and no further items were removed.

The three-factor solution revealed a clear and interpretable structure, with Factor 1 comprising nine items (9, 10, 8, 12, 2, 11, 7, 5, and 13), Factor 2 including two items (1 and 4), and Factor 3 consisting of five items (16, 14, 15, 3, and 6). All retained items demonstrated factor loadings above 0.40 on their respective factors, indicating meaningful contributions to the underlying latent constructs. The factors were interpreted based on the content of the items loading on each factor. The factor loadings are shown in Table 2.

The three-factor structure was broadly consistent with, but not identical to, the preliminary conceptual domains used during item generation. Items initially related to perceived severity, academic and social impact, family and peer health concerns, and emotional response converged into Factor 1. Items related to the perceived probability of crisis occurrence and personal infection formed Factor 2. Items concerning preparedness, protective efficacy, coping confidence, and information trust formed Factor 3. We labelled the factors based on the results of the EFA and theoretical discussions during the panel meeting. The factor labels are as follows:

**Factor 1: Perceived Impact and Personal Anxiety Related to the Crisis.** This factor comprises items that assess respondents’ perceptions of the crisis’s impact on various aspects of their lives, including work, study, personal and family health, community functioning, and psychological well-being (such as anxiety and long-term mental health). These items reflect the perceived severity and personal consequences of the crisis, as well as emotional responses like anxiety.

**Factor 2: Perceived Likelihood of Crisis Occurrence and Personal Infection.** This factor includes items related to respondents’ estimations of the probability that the crisis will occur within their community (e.g., campus) and the likelihood of their contracting a serious illness. It captures the perceived risk or vulnerability to the crisis and infection.

**Factor 3: Confidence in Preparedness, Protective Measures, and Information Trust.** This factor consists of items evaluating respondents’ perceptions of the community’s emergency preparedness (such as availability of resources and medical supplies), their confidence in personal protective behaviors (e.g., mask-wearing), and their trust and satisfaction with crisis-related information provided by the school and government. It reflects perceived efficacy and trust in both personal and institutional responses to the crisis.

#### 4.2.2. CFA Results

CFA was conducted using the WLSMV estimator for ordinal indicators. The 16-item three-factor model showed modest but interpretable fit: χ^2^ = 227.687, df = 101, *p* < 0.001; CFI = 0.891; TLI = 0.870; RMSEA = 0.067, 90% CI [0.055, 0.078]; SRMR = 0.077. The χ^2^/df value was 2.25. Although RMSEA and SRMR met the commonly recommended criteria for acceptable fit, CFI and TLI were slightly below the conventional 0.90 threshold. Therefore, the CFA results provided partial rather than strong support for the three-factor structure. The model fit indices for the CFA are shown in Table 3.

Standardized factor loadings were statistically significant for all items. Loadings ranged from 0.520 to 0.601 for Factor 1, from 0.699 to 0.855 for Factor 2, and from 0.357 to 0.631 for Factor 3. Factor 3 showed relatively modest loadings for several items, especially p3. Given this issue, additional item-deletion models were tested. Deleting p3 did not meaningfully improve overall model fit: χ^2^ = 208.518, df = 87, *p* < 0.001; CFI = 0.894; TLI = 0.872; RMSEA = 0.071, 90% CI [0.058, 0.083]; SRMR = 0.078. Deleting p15 resulted in poorer fit: χ^2^ = 214.627, df = 87, *p* < 0.001; CFI = 0.888; TLI = 0.865; RMSEA = 0.072, 90% CI [0.060, 0.085]; SRMR = 0.078. Therefore, item deletion did not produce a meaningful improvement in model fit, and the original 16-item model was retained to preserve conceptual coverage. Standardized factor loadings for each item are presented in Table 4.

### 4.3. Reliability

Internal consistency reliability was evaluated using Cronbach’s alpha. The Cronbach’s alpha coefficients for the three factors were 0.804 (Factor 1), 0.758 (Factor 2), and 0.776 (Factor 3). The overall Cronbach’s alpha coefficient for the scale was 0.779, indicating acceptable internal consistency.

## 5. Discussion

### 5.1. General Discussion of the CSPHCS

The present study developed and preliminarily validated the CSPHCS among college students, identifying a clear three-factor structure through exploratory and confirmatory factor analyses. The findings suggest that the CSPHCS may be a potentially useful instrument, although its current structure requires further refinement and validation. Compared with previous instruments developed for the general population, the CSPHCS integrates these constructs into a college-student-specific context, including campus preparedness, academic disruption, peer and family health concerns, and trust in school- and government-released information.

The quality of items is fundamental to ensuring the reliability and validity of any measurement scale. In this study, the initial item pool was developed through an integrative review of relevant literature and previously validated research instruments. To supplement this, descriptive qualitative interviews were conducted with college students to generate additional items. Following this, a panel of experts systematically reviewed and evaluated the items and indices at multiple levels, leading to revisions based on their feedback. Cognitive interviews were then employed to further refine the items, which were followed by an additional pilot test to assess their clarity and relevance. Finally, item performance was analyzed using data collected from 564 completed questionnaires, culminating in the development of a finalized 16-item scale.

The validity of the CSPHCS for public health crises was evaluated primarily from two perspectives: content validity and construct validity. In the content validity analysis, the I-CVIs and the S-CVI all met the established statistical criteria (Shi et al., 2012), which provided preliminary support for content validity.

In the EFA, all 16 items demonstrated factor loadings exceeding 0.4, indicating that each item had a meaningful and statistically significant association with its respective underlying factor. This threshold is commonly accepted in psychometric research as evidence that items adequately represent the latent constructs they are intended to measure. The extraction of three distinct factors that aligned closely with the theoretical framework further supports the construct validity of the scale.

The three-factor structure partly overlaps with previous public health risk-perception instruments and theoretical models. The first factor, Perceived Impact and Personal Anxiety Related to the Crisis, corresponds to constructs such as perceived severity, perceived consequences, worry, and affective risk perception reported in previous crisis and risk-perception scales. This factor is also consistent with the transactional model of stress and coping, which emphasizes cognitive appraisal and emotional response in stressful events. The inclusion of personal anxiety within this factor underscores the importance of affective components in shaping risk perception, consistent with prior research indicating that emotional responses can influence individuals’ behaviors and decision-making during crises (Ferrer & Klein, 2015).

The second factor, Perceived Likelihood of Crisis Occurrence and Personal Infection, is conceptually similar to perceived susceptibility in the Health Belief Model and to probability-based risk judgments in previous public health emergency instruments. It captures students’ anticipatory evaluation of both crisis occurrence and personal infection risk. This dimension emphasizes the probabilistic and anticipatory aspects of risk perception, which are critical for understanding how individuals evaluate potential threats. By combining perceptions of both crisis occurrence and personal infection risk, this factor reflects a comprehensive assessment of vulnerability that can motivate precautionary behaviors (Min et al., 2022).

The third factor, Confidence in Preparedness, Protective Measures, and Information Trust, reflects perceived efficacy, controllability, preparedness, and trust in information sources. These components are important in public health crisis contexts because trust in institutions and confidence in protective measures may influence compliance with recommended behaviors and reduce uncertainty. This factor highlights the role of external factors in shaping risk perception, as confidence in authorities and the credibility of communicated information can mitigate uncertainty and influence emotional responses. Trust in preparedness and information is particularly relevant in public health crises, where effective communication and institutional competence are essential for fostering compliance with recommended protective measures (Correia, 2024).

In the CFA, the three-factor model showed modest but interpretable fit. The use of WLSMV was appropriate because the CSPHCS items were measured using a 5-point Likert response format. The RMSEA and SRMR met commonly recommended criteria for acceptable fit, and the χ^2^/df value was within an acceptable range. However, the CFI and TLI were slightly below the conventional 0.90 threshold.

The relatively modest fit indices indicate that the current version of the CSPHCS should be regarded as a preliminary instrument rather than a fully validated measure. In particular, the third factor, Confidence in Preparedness, Protective Measures, and Information Trust, showed more heterogeneous item performance than the other two factors. This may be because the factor contains conceptually related but empirically distinguishable elements, including institutional preparedness, personal protective efficacy, coping confidence, school information timeliness, and government information trust. Although these elements are theoretically relevant to students’ crisis perception, they may not form a highly homogeneous latent dimension in the present sample. Additional item-deletion models were tested to examine whether removing low-loading items from the third factor would improve model fit. However, deleting p3 or p15 did not meaningfully improve the overall model. Moreover, removing these items would have narrowed the conceptual coverage of the factor. Therefore, the original 16-item structure was retained. Future research should further refine this factor by revising low-loading items, developing additional indicators, and testing the scale in independent and more diverse samples.

The results demonstrated that the CSPHCS possesses satisfactory content and face validity, indicating that the items are appropriate for their respective domains, as well as for the overall scale. Consensus among experts on the items was achieved. Furthermore, the reliability of the CSPHCS and each of its domains was evaluated using Cronbach’s alpha coefficients, all of which reached acceptable standards (Cortina, 1993).

### 5.2. Strengths and Limitations

A key strength of this study is the rigorous methodological approach, including a sample of college students and the use of both EFA and CFA to establish the scale’s factor structure and validity. The application of parallel analysis based on common factor analysis enhanced the accuracy of factor retention decisions.

Several limitations should be acknowledged. First, although public health crises are inherently multidimensional and can vary widely in nature and impact, this scale was developed primarily based on the recent context of the COVID-19 pandemic. As a result, the scale may not fully capture all aspects or dimensions relevant to other types of public health crises. Future research should validate and adapt the scale to encompass a broader range of public health crises to enhance its generalizability and applicability. Next, although using an online survey offers convenience and efficiency, it may lead to selection bias, compromise data validity due to unverifiable respondent identities, and increase the risk of questions being misread or answered carelessly (Singh & Sagar, 2021). Third, because the sample was drawn from a single cultural and educational context, the findings may not be generalizable to other populations or settings. In addition, although exploratory and confirmatory factor analyses were conducted on separate subsamples, both were derived from the same overall dataset and recruitment context; thus, the factor structure was internally cross-validated rather than confirmed in a fully independent sample. Finally, the CFA results showed only modest model fit. Although RMSEA and SRMR were acceptable, CFI and TLI were slightly below the conventional threshold, suggesting that the current factor structure requires further refinement.

### 5.3. Suggestions for Future Research

Future research should aim to validate the CSPHCS in diverse cultural and institutional contexts to enhance its generalizability. Longitudinal studies are recommended to explore how crisis perceptions evolve and influence behavioral and psychological outcomes. Additionally, expanding the scale by developing more items for factors with fewer indicators could improve reliability and construct coverage. Researchers might also investigate the scale’s predictive validity concerning actual health behaviors and crisis coping strategies.

### 5.4. Implications for College Professionals and Policymakers

The CSPHCS may provide a useful framework for understanding how college students perceive public health crises and how these perceptions may shape their emotional responses, preparedness, and engagement with protective behaviors. With further validation, the CSPHCS could help to identify groups of students with heightened perceived impact, anxiety, or low confidence in preparedness and information sources. Such information may inform risk communication strategies, mental health support, emergency planning, and health education programs.

The scale may also have implications for shaping students’ public health culture and attitudes. By systematically assessing students’ perceptions, college professionals can better understand how students interpret public health risks, evaluate institutional responses, and engage with collective responsibility during crises. These insights may support educational initiatives that promote evidence-based thinking, trust in credible information sources, responsible health behaviors, and a stronger sense of community participation. In this way, the application of the CSPHCS may contribute to the development of a more informed, resilient, and socially responsible student population.

At the policy level, findings generated through the CSPHCS may help inform campus-level and broader public health strategies. Combined data could be used to monitor changes in students’ crisis perceptions over time, evaluate the effectiveness of communication and preparedness interventions, and guide resource allocation for student health services. However, given the preliminary nature of the current validation results, the scale should be applied cautiously and further tested in diverse universities, cultural contexts, and public health crisis scenarios before being used for high-stakes decision-making.

## 6. Conclusions

This study developed the CSPHCS and provided preliminary evidence for its content validity, internal consistency, and three-factor structure in a sample of college students. The three factors reflected perceived impact and personal anxiety, perceived likelihood of crisis occurrence and personal infection, and confidence in preparedness, protective measures, and information trust. Future research should refine low-performing items, test the scale in independent and diverse samples, and examine test–retest reliability, convergent validity, predictive validity, and cross-cultural applicability.

## Figures and Tables

**Table 1:** Distribution of each item.

Item	Score				
	1	2	3	4	5
1. How likely do you think it is that such a crisis will occur in your community (e.g., campus) within the next year?	62 11.0%	239 42.4%	138 24.5%	95 16.8%	30 5.3%
2. If a crisis occurs, how severe do you think its impact will be on the normal operation of the community (e.g., campus), such as medical services and transportation?	26 4.6%	84 14.9%	156 27.7%	198 35.1%	100 17.7%
3. Do you think the community’s (e.g., campus) current public health emergency preparedness (such as supplies and medical resources) is sufficient?	16 2.8%	79 14.0%	173 30.7%	229 40.6%	67 11.9%
4. How likely do you estimate it is that you will contract a serious illness within the next year?	130 23.0%	228 40.4%	115 20.4%	61 10.8%	30 5.3%
5. If infected, how severe do you expect the impact on your physical health to be?	15 2.7%	78 13.8%	190 33.7%	211 37.4%	70 12.4%
6. How effective do you think the protective measures you take (such as wearing masks) are?	3 0.5%	42 7.4%	119 21.1%	258 45.7%	142 25.2%
7. How likely do you think it is that the crisis will cause an interruption to your studies?	77 13.7%	142 25.2%	158 28.0%	117 20.7%	70 12.4%
8. How severe do you estimate the impact of the crisis will be on your part-time job/internship/work?	24 4.3%	77 13.7%	124 22.0%	198 35.1%	141 25.0%
9. How do you expect the crisis to affect your learning outcomes?	15 2.7%	98 17.4%	156 27.7%	210 37.2%	85 15.1%
10. How likely do you think it is that the crisis will have a negative impact on the health of your dormitory mates/classmates?	13 2.3%	86 15.2%	154 27.3%	207 36.7%	104 18.4%
11. How likely is it that your immediate family members will face health risks due to the crisis?	37 6.6%	103 18.3%	157 27.8%	183 32.4%	84 14.9%
12. How anxious do you feel when thinking about such a crisis?	25 4.4%	82 14.5%	193 34.2%	158 28.0%	106 18.8%
13. How severe do you expect the crisis to affect your long-term mental health?	30 5.3%	114 20.2%	141 25.0%	220 39.0%	59 10.5%
14. How confident are you in your ability to cope with stress related to the crisis?	7 1.2%	68 12.1%	179 31.7%	222 39.4%	88 15.6%
15. How satisfied are you with the timeliness of crisis-related information released by the school?	11 2.0%	50 8.9%	160 28.4%	245 43.4%	98 17.4%
16. How much do you trust the crisis information released by the government?	4 0.7%	11 2.0%	81 14.4%	229 40.6%	239 42.4%

**Table 2:** Factor Loadings.

	Perceived Impact and Personal Anxiety Related to the Crisis	Perceived Likelihood of Crisis Occurrence and Personal Infection	Confidence in Preparedness, Protective Measures, and Information Trust
p9	0.619		
p10	0.585		
p8	0.582		
p12	0.575		
p2	0.560		
p11	0.516		
p7	0.507		
p5	0.502		
p13	0.473		
p1		0.707	
p4		0.637	
p16			0.484
p14			0.475
p15			0.438
p3			0.428
p6			0.428

**Table 3:** Model fit indices for the CFA.

Fit Index	Value	Reference Criterion	Interpretation
χ^2^	227.687	Smaller values preferred	Significant
χ^2^/df	2.25	<3 acceptable	Acceptable
CFI	0.891	≥0.90 acceptable; ≥0.95 good	Slightly below acceptable threshold
TLI	0.870	≥0.90 acceptable; ≥0.95 good	Below acceptable threshold
RMSEA	0.067	≤0.08 acceptable; ≤0.06 good	Acceptable
90% CI for RMSEA	0.055–0.078	Lower values preferred	Acceptable range
SRMR	0.077	≤0.08 acceptable	Acceptable

**Table 4:** Standardized Factor Loadings.

Factor	Indicator	Standardized Loading	SE	z	*p*	95% CI
Factor 1	p2	0.569	0.047	12.170	<0.001	0.478–0.661
Factor 1	p5	0.577	0.047	12.393	<0.001	0.486–0.668
Factor 1	p7	0.568	0.045	12.617	<0.001	0.480–0.656
Factor 1	p8	0.538	0.048	11.116	<0.001	0.443–0.633
Factor 1	p9	0.584	0.044	13.160	<0.001	0.497–0.670
Factor 1	p10	0.562	0.046	12.336	<0.001	0.473–0.652
Factor 1	p11	0.549	0.047	11.682	<0.001	0.457–0.641
Factor 1	p12	0.601	0.047	12.741	<0.001	0.508–0.693
Factor 1	p13	0.520	0.051	10.207	<0.001	0.420–0.620
Factor 2	p1	0.855	0.069	12.460	<0.001	0.720–0.989
Factor 2	p4	0.699	0.061	11.488	<0.001	0.580–0.818
Factor 3	p3	0.357	0.074	4.840	<0.001	0.212–0.502
Factor 3	p6	0.460	0.067	6.903	<0.001	0.329–0.590
Factor 3	p14	0.497	0.074	6.729	<0.001	0.352–0.641
Factor 3	p15	0.442	0.075	5.897	<0.001	0.295–0.589
Factor 3	p16	0.631	0.070	8.992	<0.001	0.493–0.768

## Data Availability

The data that support the findings of this study are available from the primary author upon reasonable request.
